# Editorial: Pain in infants: pain management practices and the association with outcome

**DOI:** 10.3389/fpain.2023.1216764

**Published:** 2023-06-13

**Authors:** Emma G. Duerden, Christopher McPherson

**Affiliations:** ^1^ Western Institute for Neuroscience, Western University, London, ON, Canada; ^2^ Applied Psychology, Faculty of Education, & Department of Pediatrics, Schulich School of Medicine and Dentistry, Western University, London, ON, Canada; ^3^ Department of Pharmacy, St. Louis Children’s Hospital, St. Louis, MO, United States; ^4^ Department of Pediatrics, Washington University School of Medicine, St. Louis, MO, United States

**Keywords:** analgesia, sedation, neuroprotection, developmental outcome, newborn, pain

**Editorial on the research topic** Pain in Infants: Pain Management Practices and the Association with Outcome

Critically ill infants experience a high burden of pain and agitation during intensive care due to life sustaining interventions including mechanical ventilation, surgical interventions, and routine laboratory monitoring (1). Pain has a well-documented impact on neurodevelopmental outcomes in children born before term (2, 3). A growing body of evidence informs the risks associated with major surgery (and consequent exposure to pain and anesthesia) in term infants (4). Consequently, clinicians prioritize the provision of analgesia and sedation in vulnerable infants (5). However, limited data inform the long-term impacts of commonly utilized sedatives and analgesics in preterm and term infants (6). This issue contains vital contributions to the growing body of knowledge on the intersection of pain and agitation, their treatment, and outcomes after neonatal intensive care.

Further elucidating the impact of major surgery on brain development in infants, Kagan et al. investigated the effects of early surgical interventions on the corpus callosum (CC) in infants who underwent complex perioperative critical care for long-gap esophageal atresia (LGEA), including Foker process repair. The study used non-sedated magnetic resonance imaging of preterm and term infants, as well as healthy controls, who were less than 1-year old, corrected age. The researchers segmented six sub-regions of the CC and eight sub-regions of forebrain. The findings revealed that infants who underwent LGEA repair had quantitatively smaller total CC and forebrain volumes, with sub-region II (connecting to the pre-motor cortex) being the least affected. Additionally, smaller subgenual forebrain volumes were observed, suggesting potential (mal)adaptation in limbic circuit development in this group of infants. The study recommends further investigation, including diffusion tractography studies, to better understand the global decrease in homotopic-like CC/forebrain size following complex perioperative critical care in infants with LGEA.

In the setting of mounting evidence of the neurologic impact of both minor and major procedural pain in the newborn period, the optimal interventions for common painful procedures must be defined. To contribute to the expansive body of literature on oral sucrose for minor procedural pain in preterm infants, Cavicchiolo et al. conducted a randomized, double-blind, placebo-controlled trial to evaluate the effectiveness of a second dose of sucrose during venipuncture. A total of 72 preterm infants undergoing venipuncture for routine tests were randomized into a control group [receiving a single standard dose of 24% sucrose (0.3–0.5 ml) 2 min before the procedure and a placebo 30 s after the venipuncture] and an experimental group (receiving two doses of 24% sucrose 2 min before and 30 s after the venipuncture). Pain perception was assessed at 30 s, 60 s, and 120 s after the venipuncture. The study found no significant difference in pain perception between the two groups at any of the time points evaluated. Therefore, a second dose of sucrose during venipuncture in preterm infants is not recommended based on the findings of this study. A single dose of oral sucrose is clearly beneficial to newborns requiring venipuncture, heel lance, or intramuscular injections (7). However, there is concern regarding the long-term impact of repetitive dosing (8). In this setting, reinforcement of the efficacy of a single dose provides valuable evidence to clinicians. Combined with recent dose finding studies, these results contribute to a vital goal in this arena—providing effective analgesia while simultaneously minimizing exposure to agents with neurologic effects (9).

A subset of painful procedures in infants warrant opioid analgesia. In infants without intravenous access, clinicians must accept the delayed onset of erratically absorbed oral agents or perform urgent procedures without analgesia. Pediatric emergency departments have pioneered the use of intranasal (IN) sedation and/or analgesia (10); this represents an emerging option in neonatal intensive care. Contributing vital evidence to support this practice, Cheng et al. evaluated the effectiveness and safety of IN fentanyl for procedural pain management in preterm infants. The retrospective cohort study was conducted in an academic neonatal intensive care unit and included 13 infants who received IN fentanyl on 22 occasions between May 2019 and December 2020. Painful procedures included lumbar puncture, central venous catheter insertion, and endotracheal intubation. The main outcome measures were pain responses, physiological parameters before and up to 60 min after IN fentanyl administration, and adverse events. The results showed a significant difference in the mean pre- and post-procedure pain scores, as measured by the Premature Infant Pain Profile, with a decrease in pain scores after IN fentanyl administration (5.4 vs. 4.2, *p* = 0.04). There were no significant differences in physiological parameters before and after administration. Two adverse events, one apnea and one desaturation, were noted. The study suggests that IN fentanyl may be considered as an alternative pharmacotherapy for procedural pain management in preterm infants when intravenous access is not available, based on the limited experience in this study. This retrospective study provides a model for other neonatal intensive care units utilizing intranasal analgesia to track and report their outcomes, or reassures clinicians reluctant to extrapolate from older pediatric populations.

Clinicians must understand the acute effects of analgesics in preterm infants before prescribing these agents. Uncovering the long-term implications of analgesia during a prolonged neonatal hospitalization continues to challenge researchers. To extend our knowledge of the potential impacts of the synthetic opioid fentanyl, Mills et al. evaluated the association between the cumulative fentanyl exposure of very preterm infants during neonatal intensive care and 5-year neurodevelopmental and socioemotional outcomes. Eighty-four children born at or before 30 weeks gestational age between April 2007 and June 2010 were followed longitudinally from birth to 5 years of age. Despite a correlation with cerebellar injury and growth at term equivalent age in this cohort, cumulative fentanyl dose did not correlate with 5-year neurodevelopmental outcomes when controlling for confounders (11). Both cumulative fentanyl dose and family dysfunction were associated with caregiver reports of socioemotional problems after adjustment for covariates. These findings highlight the paucity of data informing the long-term impact of analgesics and sedatives commonly utilized in neonatal intensive care. Collaborative research comparing clinical strategies and outcomes across centers should be prioritized, in a setting where large randomized controlled trials of chronic analgesia/sedation may not be palatable to clinicians or funding organizations (12).

Randomized controlled trials of chronic sedation in this area of research are daunting, but not impossible. Finally in this issue, Baserga et al. present the framework of an ongoing phase II safety and pharmacokinetic randomized controlled trial comparing dexmedetomidine and morphine for sedation during therapeutic hypothermia in neonates greater than or equal to 36 weeks gestational age. Therapeutic hypothermia saves lives and reduces disability in late preterm and term neonates with moderate or severe encephalopathy after birth. However, disability in survivors remains too common. The appropriate treatment of agitation during therapeutic hypothermia may improve outcomes, but also may cause adverse effects (respiratory depression, feeding intolerance, neuronal apoptosis). Worldwide practice is variable, with common practices including no sedation, morphine, and dexmedetomidine. To inform future comparative effectiveness research, this group from University of Utah will enroll 50 neonates in the Dexmedetomidine Use in Infants Undergoing Cooling Due to Neonatal Encephalopathy (DICE) trial. The primary goals of the trial are to evaluate the safety of dexmedetomidine relative to the more commonly utilized morphine, collect pharmacokinetic data to optimize dexmedetomidine dosing, and document the feasibility of evaluating long-term neurodevelopmental outcomes in a future large trial. The trial has enrolled one quarter of their goal study population, and the field eagerly anticipates results to inform both clinical practice and future research efforts in this domain.

## Conclusions

The articles included in this collection provide insights and future directions in key domains of neonatal pain research, including the implications of major surgery, treatment approaches to pain, and the long-term implications of continuous analgesia in both preterm and term infants. These articles may contribute to important clinical practice changes in neonatal intensive care units. More likely, they will inform ongoing research in this challenging field using the methodologies of both retrospective observational studies and prospective randomized trials leveraging early informants of long-term outcome like magnetic resonance imaging before diligently tracking and evaluating childhood outcomes in survivors. We will eagerly follow the important work of contributors to this issue and others in the field to inform compassionate neonatal care that optimizes long-term outcomes.
